# Characterization and analysis of full-length transcriptomes from two grasshoppers, *Gomphocerus licenti* and *Mongolotettix japonicus*

**DOI:** 10.1038/s41598-020-71178-5

**Published:** 2020-08-26

**Authors:** Hao Yuan, Xue Zhang, Lina Zhao, Huihui Chang, Chao Yang, Zhongying Qiu, Yuan Huang

**Affiliations:** 1 College of Life Sciences, Shaanxi Normal University, Xi’an, 710062 China; 2 Shaanxi Institute of Zoology, Xi’an, China; 3 School of Basic Medical Sciences, Xi’an Medical University, Xi’an, China

**Keywords:** Molecular biology, Transcriptomics, Functional genomics

## Abstract

Acrididae are diverse in size, body shape, behavior, ecology and life history; widely distributed; easy to collect; and important to agriculture. They represent promising model candidates for functional genomics, but their extremely large genomes have hindered this research; establishing a reference transcriptome for a species is the primary means of obtaining genetic information. Here, two Acrididae species, *Gomphocerus licenti* and *Mongolotettix japonicus*, were selected for full-length (FL) PacBio transcriptome sequencing. For *G. licenti* and *M. japonicus*, respectively, 590,112 and 566,165 circular consensus sequences (CCS) were generated, which identified 458,131 and 428,979 full-length nonchimeric (FLNC) reads. After isoform-level clustering, next-generation sequencing (NGS) short sequences were used for error correction, and remove redundant sequences with CD-HIT, 17,970 and 16,766 unigenes were generated for *G. licenti* and *M. japonicus*. In addition, we obtained 17,495 and 16,373 coding sequences, 1,082 and 813 transcription factors, 11,840 and 10,814 simple sequence repeats, and 905 and 706 long noncoding RNAs by analyzing the transcriptomes of *G. licenti* and *M. japonicus*, respectively, and 15,803 and 14,846 unigenes were annotated in eight functional databases. This is the first study to sequence FL transcriptomes of *G. licenti* and *M. japonicus*, providing valuable genetic resources for further functional genomics research.

## Introduction

One important goal of functional genomics is to establish relationships between genotypes and phenotypes based on genomic sequence information and various omics techniques^1^. The rapid development of high-throughput sequencing technology has greatly facilitated the study of functional genomics, especially the completion of genome sequencing of a large number of species ^2–5^. However, genome assembly is difficult in species with large genomes, especially those with high heterozygosity and regions with high repeat content^6^. Overall, genomic approaches to genotype–phenotype association in species with large genomes still face major challenges.

Transcriptomics focuses on the transcribed portion of the genome by sequencing cDNA rather than genomic DNA, thus reducing the size of the sequencing target space, and can be viewed as an alternative to genomic approaches^7^. Furthermore, a unique feature of transcriptomics is that it can quantify changes in expression level for each gene among different transcriptome samples. As a low-cost next-generation sequencing (NGS) technology, RNA sequencing (RNA-seq) has become a mainstream tool for studying transcriptomics^8^. At present, RNA-seq is widely used not only for gene expression profiling, genome annotation and noncoding RNA prediction and quantification but also to gain deep insight into the level of gene expression, the structure of genomic loci, and the sequence variation present at loci (e.g., SNPs)^7^. RNA-seq has revolutionized the field of transcriptomics and improved our understanding of genome expression and regulation.

Although RNA-seq has been applied to a large number of species and studies, short-read (e.g., reads obtained from Illumina sequencing platforms) sequencing does not provide full-length (FL) transcript sequences due to its inherent length limitations, thereby limiting its utility. Moreover, differences in transcript abundance and the presence of different isoforms have greatly hampered transcriptome assembly^9^. Third-generation sequencing (TGS) technology, as represented by PacBio (Pacific Biosciences) single molecule real-time (SMRT) sequencing technology, can help overcome the limitations of short-read sequences by providing FL transcripts directly, without further assembly^10^. Read length is an important advantage of PacBio sequencing. The read length generated by PacBio RSII system ranged from 5 to 60 kb, with an average length around 12 kb, and the newly-released PacBio Sequel sequencer can generate reads longer than 20 kb on average, which is about 200 times longer than those reads generated by conventional NGS instruments^11^. Due to the much longer read lengths of PacBio sequencing, the precise locations and sequences of repetitive regions and isoforms can often be resolved with a single read.

Grasshoppers from the family Acrididae (Orthoptera, Caelifera) are widely distributed, easy to collect and important in agriculture, and their classification has always been at the forefront of insect taxonomy^12–14^. Moreover, the Acrididae fauna of most regions is well known, and a worldwide taxonomic file is available (https://Orthoptera.SpeciesFile.org)^15^; these features make Acrididae a good subject for the study of phylogeny and evolution. Acrididae also exhibits many important genetic characteristics and has a promising future in the study of the genetics of sexual ornamentation, maintenance of color polymorphism, genome size evolution, etc.^16^. However, due to the large genome of Acrididae (known Acrididae genome sizes vary from 3.76 Gb for *Melanoplus differentialis* to 16.56 Gb for *Podisma pedestris*; data from Genome Size Database: www.genomesize.com), their genomic information is lacking. Only for the migratory locust *Locusta migratoria* (genome size is ~ 6.5 Gb) is a complete genome sequence available thus far^17^.

In recent years, due to the development of NGS technology, an increasing number of Acrididae transcriptome analyses have appeared, and such reports potentially provide resources for advancing functional genomics research in Acrididae^16,18–20^. However, the read length generated by NGS technology is too short to capture entire transcripts. In addition, short-read sequencing is incapable of annotating and quantifying transcriptomes on the level of RNA transcript isoforms, which hamper further studies of alternative splicing (AS) forms, alternative polyadenylation (APA) events and fusion transcripts^21^. PacBio sequencing overcomes the limitations of short sequence reads by generating sequencing reads of kilobase size; these longer reads can also help improve our understanding of RNA processing^22^.

*Gomphocerus licenti* (Chang, 1939), also named the club-legged grasshopper (Orthoptera, Caelifera, Acridoidea, Acrididae), has intriguing features , including the striking sexual dimorphism of its foreleg morphology and a widespread green–brown polymorphism of body color. However, this species has limited molecular resources; the only information available to date is its mitochondrial genome. *Mongolotettix japonicus* (Bolívar, 1898) also belongs to Orthoptera, Caelifera, Acridoidea and Acrididae, and its main characteristic is that the body lengths and wing forms of male and female individuals have significant sexual dimorphism. The body length of the male of *M. japonicus* is 16.5 ~ 18.0 mm, and the forewings are relatively developed, reaching 4/5 of the hind femur, while the female is 26.0 ~ 27.0 mm, with the forewings scaly and the apex reaching the middle of the second abdominal tergite^23^. The molecular resources of *M. japonicus* are even fewer: so far, only a 658 bp mitochondrial genome cytochrome oxidase subunit I (COI) sequence has been published on NCBI.

Here, we reported the complete and FL transcriptomes of *G. licenti* and *M. japonicus* by combining PacBio sequencing and RNA-seq, which will serve as a reference resource for gene functional studies. Based on the obtained FL transcriptome datasets, we performed transcription factor (TF) prediction, simple sequence repeat (SSR) analysis, long noncoding RNA (lncRNA) prediction and transcript functional annotation. These studies might be a valuable resource for further investigation of *G. licenti* and *M. japonicus*.

## Results

### The full-length transcriptome sequences of *G. licenti* and *M. japonicus*

To identify as many transcripts as possible, high-quality RNA samples were extracted from three adult females and three adult males of *G. licenti* and then pooled together in equal amounts for library preparation and sequencing. A total of 34.16 Gb clean data were generated from PacBio sequencing, which yielded 590,112 circular consensus sequence (CCS) reads with a mean length of 3,107 bp. By searching for the presence of poly-A tails and the 5′ and 3′ primers, 458,131 full-length nonchimeric (FLNC) reads and 131,027 non-full-length (NFL) reads were further identified from the CCS reads. After isoform-level clustering based on the iterative clustering for error correction (ICE) algorithm and polishing based on the Arrow algorithm, a total of 29,340 polished FL consensus isoforms with an average length of 2,995 bp were generated from the FLNC reads, including 28,736 high-quality (HQ; accuracy ratio > 99%) and 601 low-quality (LQ; accuracy ratio ≤ 99%) sequences (Table 1). Furthermore, 41.46 Gb clean reads were generated after adaptor sequence trimming and LQ read filtering in an Illumina platform (Additional file 1: table S1). These clean reads were subsequently used to correct the low-quality isoforms from PacBio sequencing by Proovread v2.12 (https://github.com/BioInf-Wuerzburg/proovread) ^24^ software (Additional file 1: table S2). After redundancy removal via the CD-HIT program and filtering reads less than 200 bp in length, the consensus isoforms were finally clustered into a total of 17,932 unigenes for subsequent analysis (Table 1). The final unigene sequences have been deposited at DDBJ/EMBL/GenBank by Transcriptome Shotgun Assembly project under the accession GICA00000000. The version described in this paper is the first version, GICA01000000.

**Table 1:** Summary for the full-length transcriptome of *G. licenti* and *M. japonicus* using PacBio sequencing.

Parameters	*G. licenti*	*M. japonicus*
**Sequencing data**		
Number of clean data (Gb)	34.16	34.55
Number of CCS	590,112	566,165
Read bases of CCS	1,833,944,798	1,774,198,050
Mean read length of CCS (bp)	3,107	3,133
Number of full-length non-chimeric reads	458,131	428,979
Number of non-full-length reads	131,027	136,304
Number of filtered short reads	954	882
Full-length non-chimeric percentage (%)	77.63	75.77
**Isoform clustering**		
Number of consensus isoforms	29,340	25,379
Mean read length of consensus isoforms (bp)	2,995	2,943
Number of polished high-quality isoforms	28,736	24,831
Number of polished low-quality isoforms	601	544
Percent of polished high-quality isoforms(%)	97.94	97.86
**Unigene**		
Number of unigenes	17,932	16,739
Mean read length (bp)	3,000	2,933
Smallest read length (bp)	200	200
Largest read length (bp)	14,592	12,710
N50 length (bp)	3,605	3,503
GC content (%)	42.2	42.6

The same method was used for the FL transcriptome analysis of *M. japonicus*, for which all the results of PacBio sequencing are shown in Table 1, and the final unigene sequences are available in DDBJ/EMBL/GenBank under the accession GIBZ00000000. The version described in this paper is the first version, GIBZ01000000. The Illumina results of *M. japonicus* are presented in Additional file 1: Table S1.

To test the completeness of the two FL transcriptomes, Benchmarking Universal Single-Copy Orthologs (BUSCO) v3.0.2 with the Insecta odb9 database^25^ was used. For *G. licenti*, the BUSCO results showed a completeness score of 70.4%, a fragmentation score of 2.7% and a missing score of 26.9% (Additional file 1: table S3). For *M. japonicus*, 68.8% of BUSCOs were complete, 3.1% were fragmented and 28.1% were missing (Additional file 1: table S3).

### De novo assembly from Illumina short reads

The transcriptomes of three female and three male of *G. licenti* and *M. japonicus* were separately sequenced using the Illumina HiSeq X Ten platform. After trimming and filtering, a total of 41.46 Gb and 40.81 Gb clean reads were obtained from *G. licenti* and *M. japonicus*, respectively. Based on these clean reads, 53,453 unigenes of *G. licenti* and 53,652 unigenes of *M. japonicus* were de novo assembled with Trinity v2.5.1 software^26^, and their N50 length was 2,047 bp and 2,074 bp, respectively. For more information, see Additional file 1: table S4.

### Comparison between PacBio unigenes and Illumina unigenes

**Figure 1:**
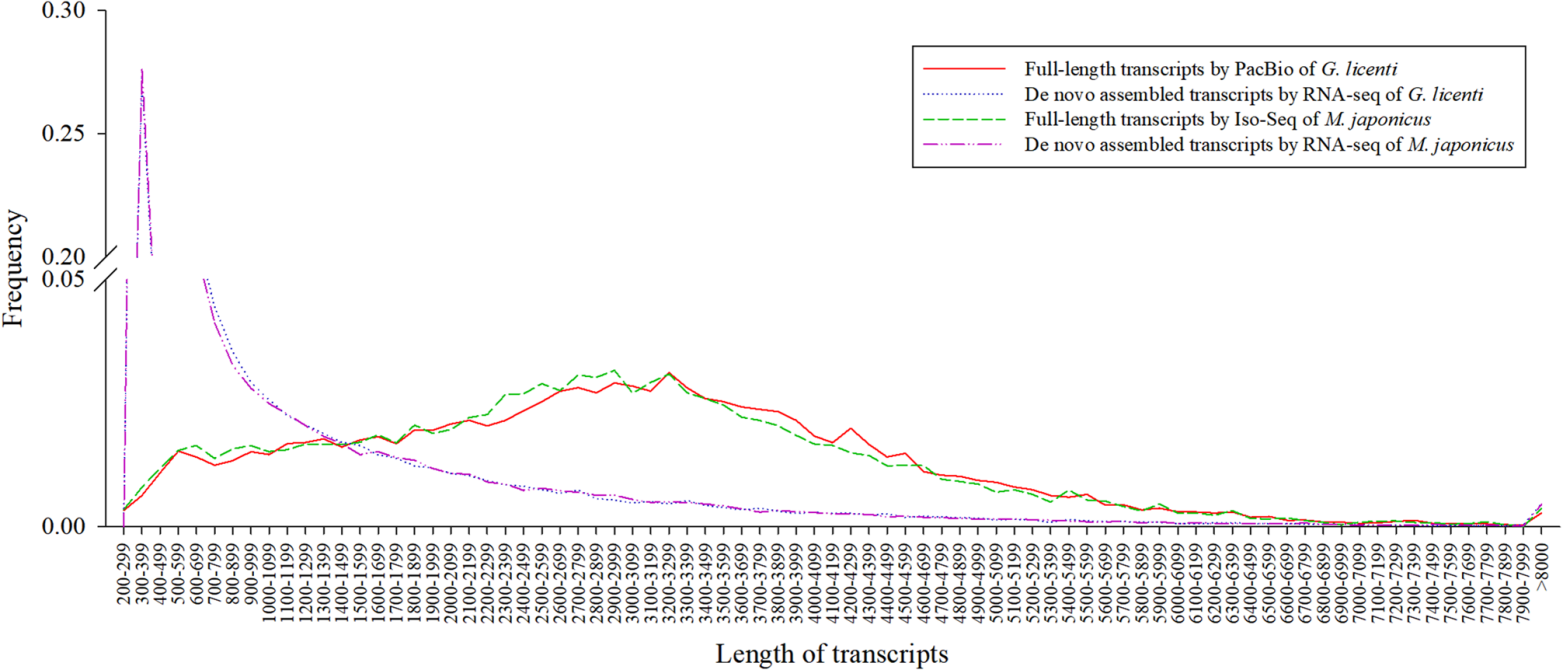
The comparison of unigene length distributions between pacbio sequencing and Illumina sequencing.

Both the mean and N50 lengths of PacBio unigenes were obviously longer than Illumina unigenes in both *G. licenti* and *M. japonicus* (Table 1 and Additional file 1: table S4). Most of PacBio unigenes of *G. licenti* and *M. japonicus* had lengths > 1,500 bp, accounting for 82.67% and 81.82% of the total number, while most Illumina unigenes of *G. licenti* and *M. japonicus* had lengths < 1,500 bp, accounting for 76.40% and 76.21%, respectively (Fig. 1).

BLAST v2.2.31 software^27^ was used to compare Illumina unigenes and PacBio unigenes with the parameter set to -e 1e-5. In *G. licenti* and *M. japonicus*, only 7,692 (14.39%) and 7,027 (13.10%) of Illumina unigenes were highly similarity to 62.41% and 63.52% of PacBio unigenes, respectively (Additional file 1: table S5). In *G. licenti*, 6,740 PacBio unigenes were no blast hit Illumina unigenes, of which 81.10% were annotated to the NR database, and 45,761 Illumina unigenes were no blast hit PacBio unigenes, of which 36.45% were annotated to the NR database. In *M. japonicus*, the no blast hit unigenes of PacBio sequencing and Illumina sequencing were 6,107 and 46,625, with the proportion of NR annotation were 85.43% and 37.06%, respectively.

In addition, we also mapped PacBio unigenes and Illumina unigenes to the genome sequences of *L. migratoria* using Minimap2 (v2.15; https://github.com/lh3/minimap2)^28^. The results showed that the mapping ratio of PacBio unigenes of *G. licenti* and *M. japonicus* was obviously higher than Illumina unigenes (Additional file 1: table S5).

### Open reading frame and transcription factor prediction

**Figure 2:**
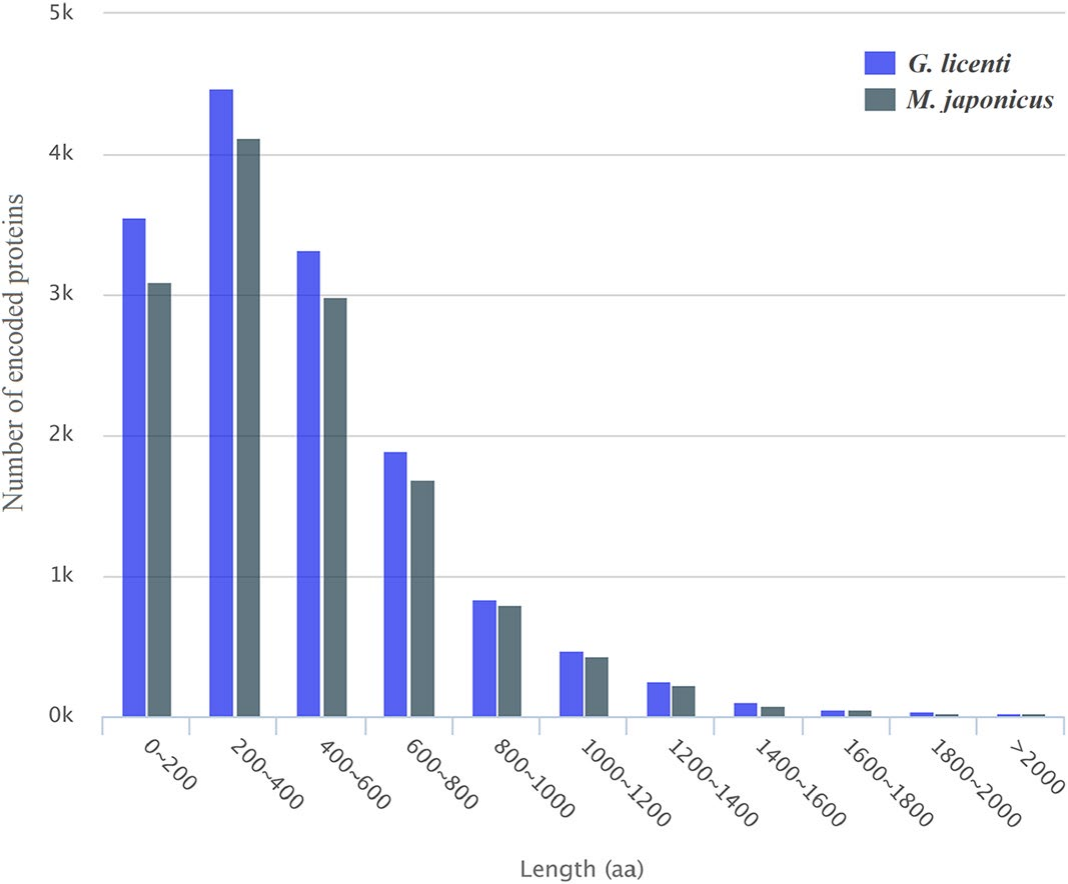
The length distributions of the complete encoded protein sequences of *G. licenti* and *M. japonicus*.

FL open reading frame (ORF) sequences were identified, and the corresponding encoded protein sequences were predicted by TransDecoder v5.0.1 (https://github.com/TransDecoder/TransDecoder/)^29^. In total, 17,495 ORFs were predicted from *G. licenti*, including 15,031 (85.9%) complete ORFs, of which 12,263 (81.6%) complete ORFs had homologous entries in the Pfam database (Additional file 2: table S6). A total of 16,373 ORFs were predicted from *M. japonicus*, of which 13,576 were complete ORFs, and of the complete ORFs, those with homologous entries in the Pfam database accounted for 84.1% (Additional file 2: table S7). The length distribution of the protein sequences encoded by the complete ORFs of *G. licenti* and *M. japonicus* is shown in Fig. 2; the lengths of the *G. licenti* ORFs ranged from 41 to 2,700 aa with an average length of 443 aa, and the lengths of the *M. japonicus* ORFs ranged from 40 to 3,709 aa with an average length of 448 aa.

**Figure 3:**
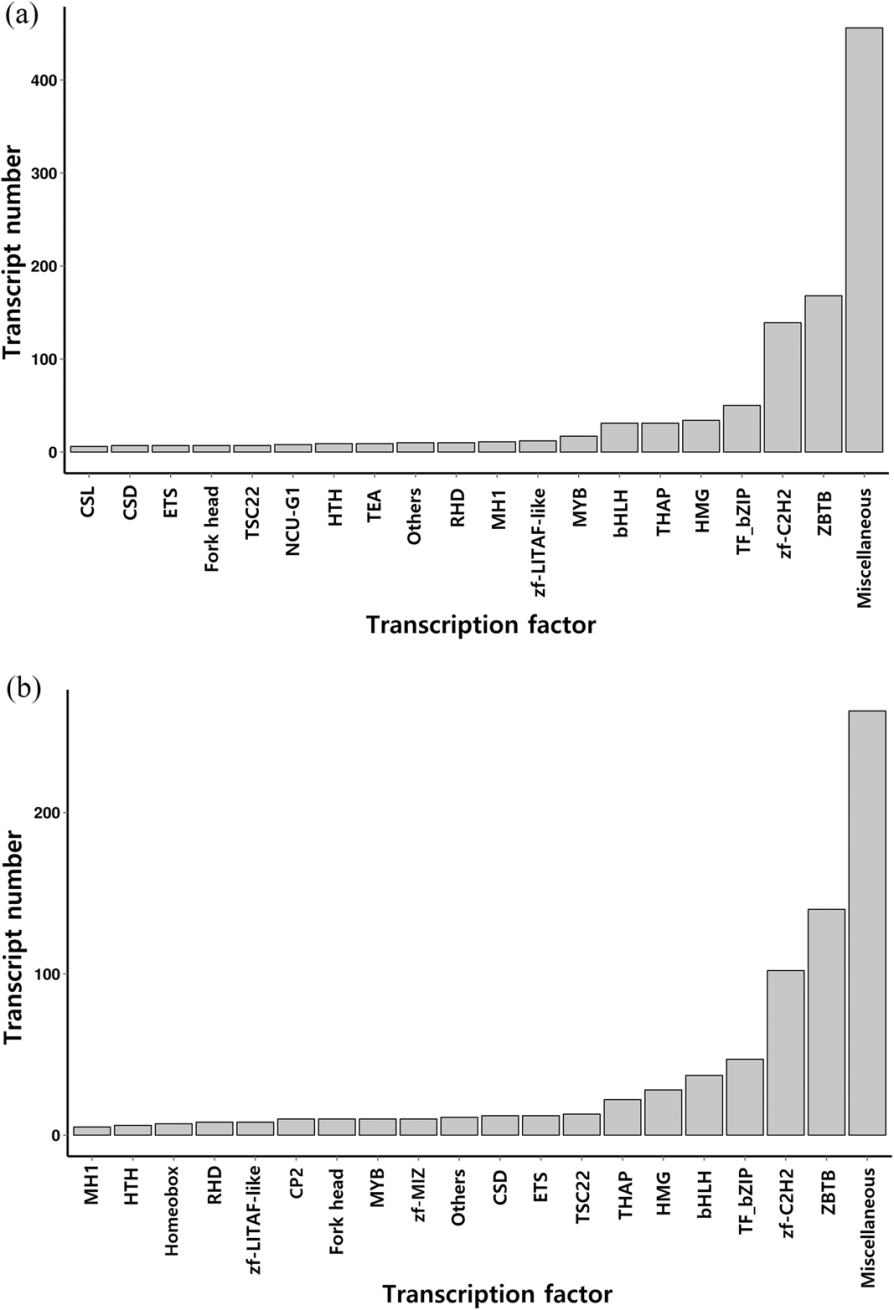
Classification of the top 20 TF families. (**a**) *G. licenti*. (**b**) *M. japonicus*.

TFs regulate gene transcription by modulating the binding of RNA polymerase to the DNA template, and thus, they play important regulatory roles in animal growth and development. In this study, 1,082 putative TFs from 41 TF gene families were identified in *G. licenti* (Additional file 2: table S8), and 813 putative TFs from 39 TF gene families were identified in *M. japonicus* (Additional file 2: table S9). The top 20 TF families of *G. licenti* and *M. japonicus* are shown in Fig. 3.

### Simple sequence repeat and long noncoding RNA identification

**Figure 4:**
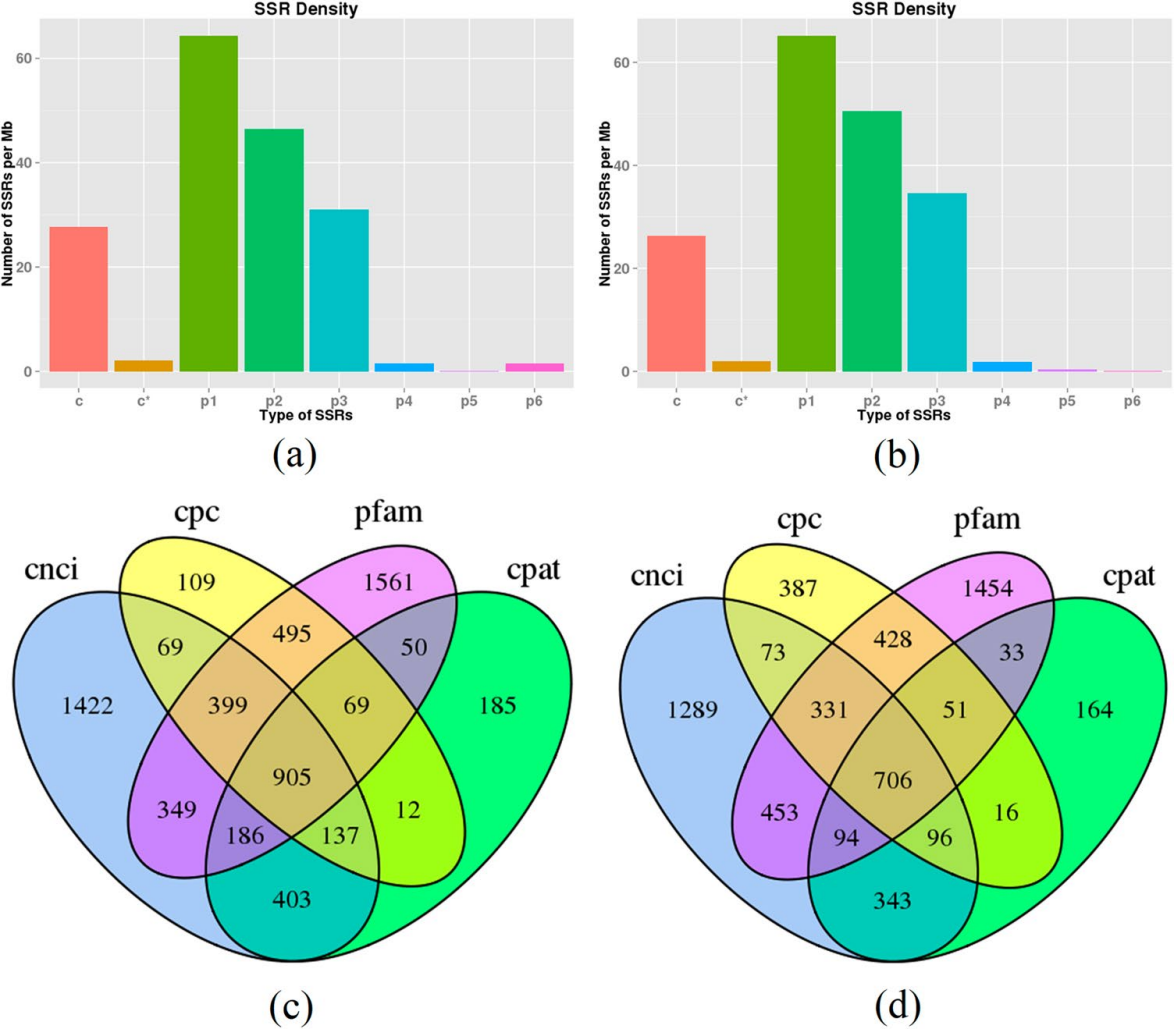
Densities of different types of SSRs and Venn diagrams of the numbers of lncRNAs identified by CPC, CNCI, Pfam and CPAT. (**a**) Densities of different types of SSRs in *G. licenti*. (**b**) Densities of different types of SSRs in *M. japonicus*. (**c**) Venn diagram showing numbers of identified lncRNAs in *G. licenti*. (**d**) Venn diagram showing numbers of identified lncRNAs in *M. japonicus*.

The MIcroSAtellite identification tool (MISA)^30^ was used for SSR analysis of the unigenes with lengths greater than 500 bp. For *G. licenti*, 17,566 unigenes were subjected to SSR analysis, and a total of 11,840 SSRs were identified in 6,436 unigenes, of which 2,785 unigenes contained more than one SSR and 2,467 SSRs were present in compound form (Additional file 2: table S10). For *M. japonicus*, a total of 10,814 SSRs were identified in 16,351 unigenes for SSR analysis, of which 2,517 contained more than one SSR and 1,963 existed in a compound form (Additional file 2: table S10). In both species, mono-, di-, and trinucleotide repeats were the most abundant SSRs, accounting for 35.3%, 38.5%, and 23.4% of all SSRs in *G. licenti* and 35.0%, 39.8% and 23.2% of all SSRs in *M. japonicus*, respectively. The densities of different types of SSRs are listed in Fig. 4a,b.

LncRNA is another important component of the transcriptome. To identify lncRNAs in the PacBio data, four analysis methods, including Coding Potential Calculator (CPC)^31^, Coding-Non-Coding Index (CNCI)^27^, Coding Potential Assessment Tool (CPAT)^32^ and Pfam protein structure domain analysis, were used. In total, 905 and 706 lncRNAs were predicted from *G. licenti* and *M. japonicus*, respectively, by all four methods (Fig. 4c,d). By filtering transcripts < 300 bp in length, 829 transcripts were confirmed as lncRNAs in *G. licenti* and 632 transcripts as lncRNAs in *M. japonicus*.

### Alternative splicing analysis

A total of 605 and 594 AS events were identified from *G. licenti* and *M. japonicus,* respectively. The AS results of *M. japonicus* were shown in Additional file 2: table S11, and of *G. licenti* were shown in Additional file 2: table S12. Additionally, since there was no available reference genome for *G. licenti* and *M. japonicus*, we could not determine the types of AS events.

### Functional annotation

**Figure 5:**
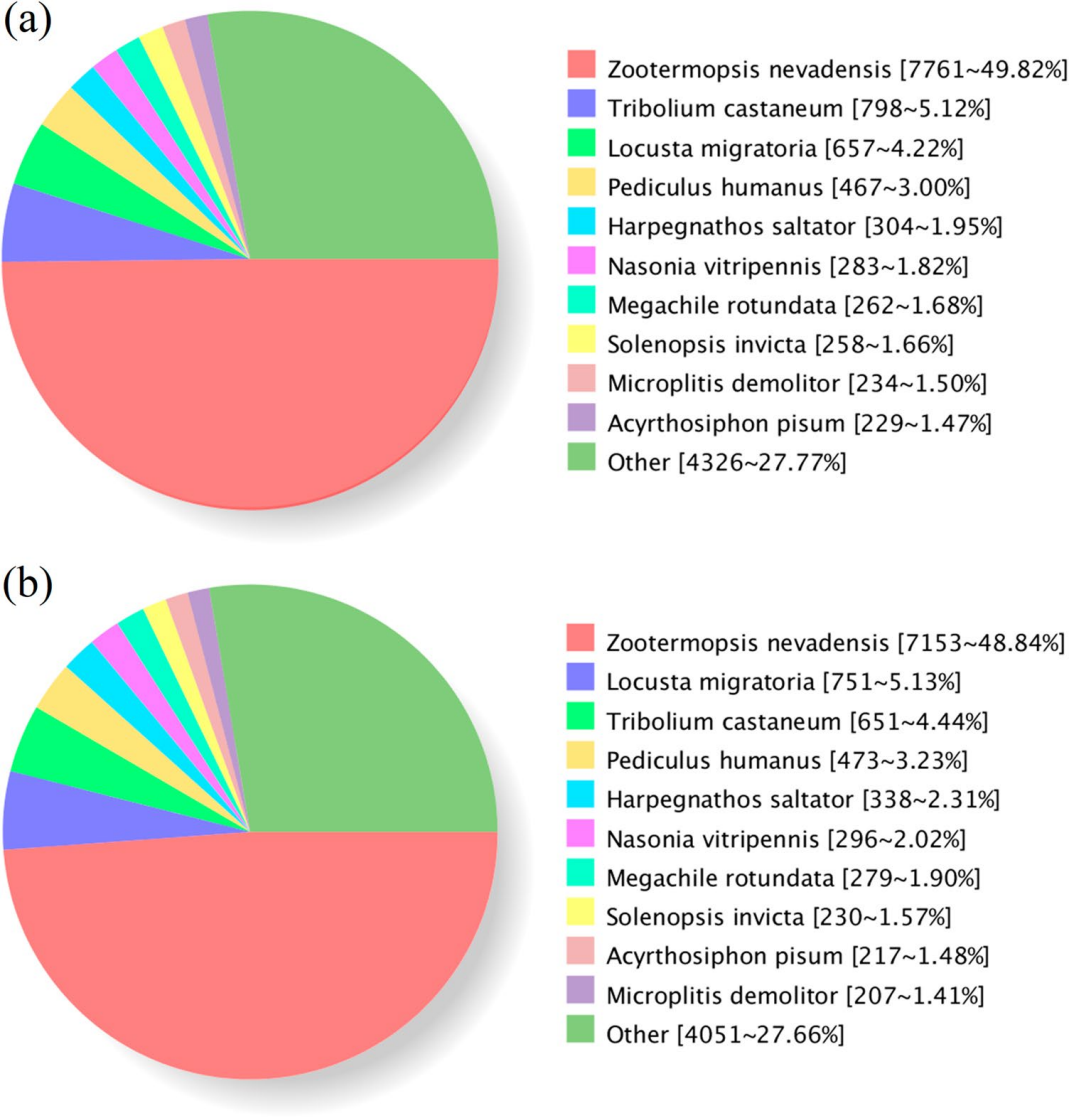
Distribution diagram of species containing homologous sequences in NR. (**a**) *G. licenti*. (**b**) *M. japonicus*.

A total of 17,970 unigenes from *G. licenti* and a total of 16,766 unigenes from *M. japonicus* were functionally annotated by searching the NR (NCBI nonredundant protein sequences), Swiss-Prot (a manually annotated and reviewed protein sequence database), KOG/COG/eggNOG (Clusters of Orthologous Groups of proteins), GO (Gene Ontology), KEGG (Kyoto Encyclopedia of Genes and Genomes)^33^ and Pfam (Protein family) databases. In total, 15,803 (87.94%) and 14,846 (88.55%) unigenes were successfully annotated in *G. licenti* and *M. japonicus*, respectively (Table 2 and Additional file 2: table S13 and table S14). By querying the NR database, we analyzed the species containing the identified homologous sequences and found that the largest number of unigenes in *G. licenti* (7,761, 49.82%) and *M. japonicus* (7,153, 48.84%) belonged to *Zootermopsis nevadensis* (Fig. 5). In *G. licenti*, the next largest numbers of unigenes were found in *Tribolium castaneum* (798, 5.12%), followed by *L. migratoria* (657, 4.22%), *Pediculus humanus* (467, 3.00%) and *Harpegnathos saltator* (304, 1.95%) (Fig. 5a), but in *M. japonicus*, the next largest numbers of unigenes were found in *L. migratoria* (751, 5.13%), followed by *T. castaneum* (651, 4.44%), *P. humanus* (473, 3.23%) and *H. saltator* (338, 2.31%) (Fig. 5b).

**Table 2:** Annotation of full-length transcript datasets to public databases.

Annotated databases	*G. licenti*		*M. japonicus*	
	Unigene number	Percentage (%)	Unigene number	Percentage (%)
COG	6,108	33.99	5,724	34.14
GO	8,065	44.88	7,637	45.55
KEGG	8,115	45.16	7,338	43.77
KOG	11,921	66.34	11,246	67.08
Pfam	13,686	76.16	13,046	77.81
Swiss-Prot	11,179	62.21	10,717	63.92
EggNOG	15,059	83.80	14,238	84.92
NR	15,594	86.78	14,669	87.49
All annotated	15,803	87.94	14,846	88.55
All analysed	17,970	100.00	16,766	100.00

**Figure 6:**
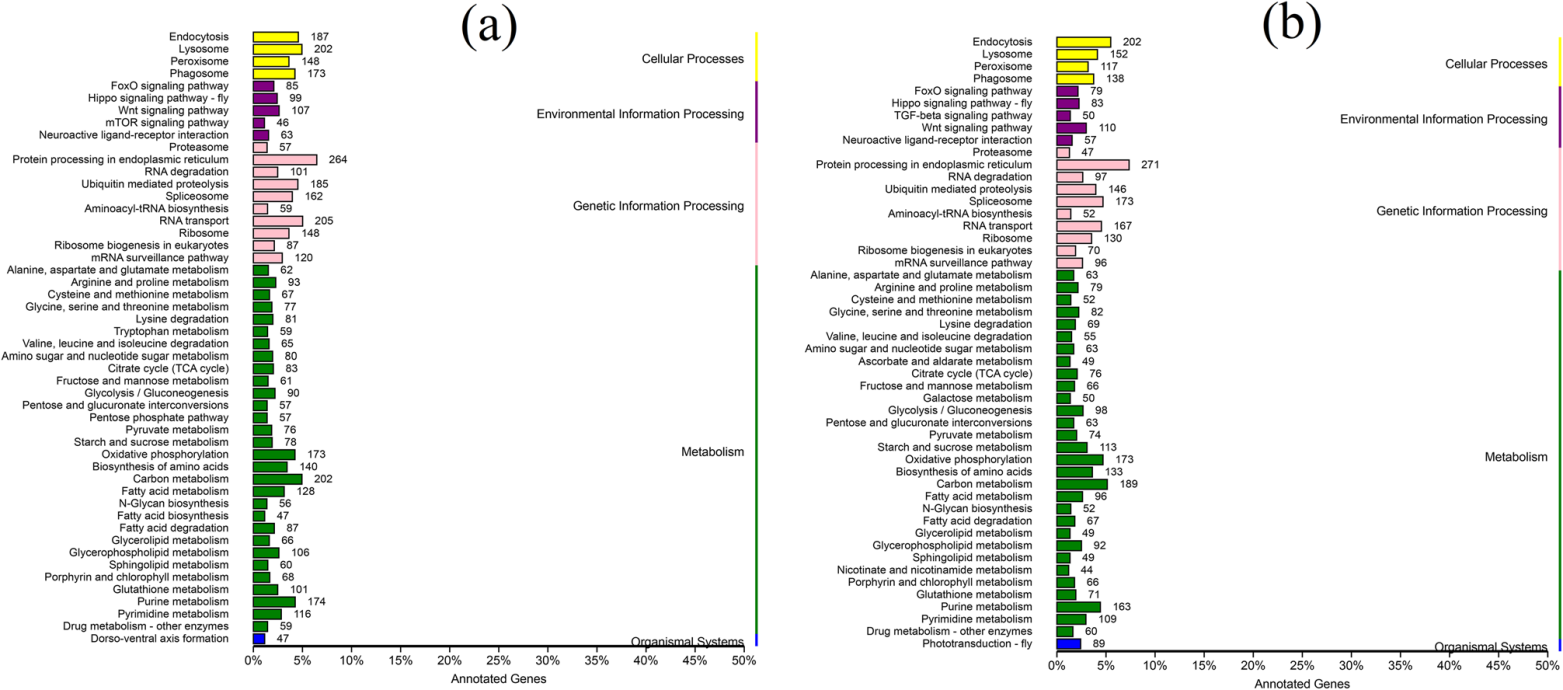
KEGG pathway classifications (Kanehisa, M. & Goto, S., 2000) for all annotated unigenes. (**a**) *G. licenti*. (**b**) *M. japonicus*.

For *G. licenti*, KEGG analysis revealed that the 8,115 annotated unigenes were assigned to 209 biological pathways, and the top five most annotated KEGG pathways were “Protein processing in endoplasmic reticulum” (264 unigenes), “RNA transport” (205 unigenes), “Carbon metabolism” (202 unigenes), “Lysosome” (202 unigenes) and “Endocytosis” (187 unigenes) (Fig. 6a and Additional file 2: table S15). For *M. japonicus*, 7,338 unigenes were annotated to 211 KEGG pathways, and “Protein processing in endoplasmic reticulum” (271 unigenes), “Endocytosis” (202 unigenes), “Carbon metabolism” (189 unigenes), “Oxidative phosphorylation” (173 unigenes) and “Spliceosome” (173 unigenes) were the top five KEGG pathways annotated (Fig. 6b and Additional file 2: table S16). Among these pathways, 83 unigenes of *G. licenti* and 72 unigenes of *M. japonicus* were assigned to “xenobiotic biodegradation and metabolism”, which related to pesticide degradation, and contained three major subcategories: “Drug metabolism—cytochrome P450” (ko00982), “Drug metabolism—other enzymes” (ko00983), and “Metabolism of xenobiotics by cytochrome P450” (ko00980). Furthermore, many unigenes from *G. licenti* and *M. japonicus* that were associated with the KEGG pathways Wnt (ko04310), Notch (ko04330), Hippo (ko04390), TGF-beta (ko04350), JAK-STAT (ko04630), MAPK-fly (ko04013), and Hedgehog (ko04340) were also found. These pathways play crucial role in the growth and development of insects^34^.

**Figure 7:**
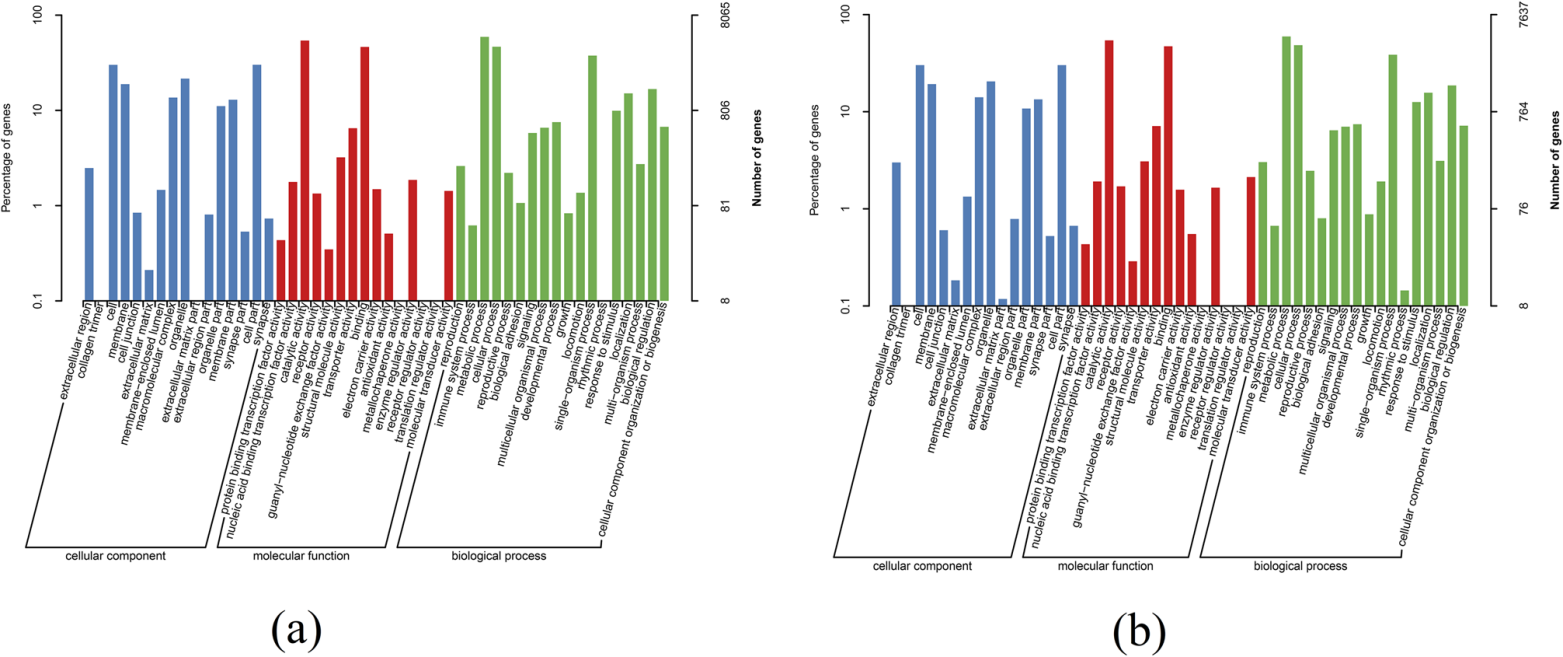
Distribution of GO terms for all annotated unigenes. (**a**) *G. licenti*. (**b**) *M. japonicus*.

As one unigene could correspond to multiple GO categories, the annotated 8,065 unigenes of *G. licenti* were classified into at least one of the three main GO categories: 3,509 (43.51%) were classified as cellular component, 6,842 (84.84%) as molecular function and 5,193 (64.39%) as biological process (Fig. 7a and Additional file 2: table S17). A total of 7,637 M*. japonicus* unigenes were annotated to GO categories, and the largest category was molecular function (6,466, 84.67%), followed by biological process (5,082, 66.54%) and cellular component (3,365, 44.06%) (Fig. 7b and Additional file 2: table S18). Notably, in the biological process category, we found that 210 genes of *G. licenti* and 231 genes of *M. japonicus* were related to reproduction, 178 genes of *G. licenti* and 188 genes of *M. japonicus* were related to reproductive process, 607 genes of *G. licenti* and 567 genes of *M. japonicus* were related to developmental process, and both species had 67 genes related to growth.

## Discussion

A good foundation for taxonomic and phylogenetic research exists in Acrididae, especially given the publication, in recent years, of a large number of mitochondrial genome sequences, which provide abundant molecular resources for molecular phylogenetic study of the family^14,35–39^. At the same time, Acrididae are extremely diverse in terms of size, body shape, feeding biology, ecology, geographical distribution and life-history traits^12,40,41^, and thus, this family has high potential to provide model candidates for functional genomics research. Although Acrididae are favored by many researchers for these reasons^42–45^, the lack of genome and transcriptome information has seriously hindered functional genomics studies of Acrididae. With the development of NGS technology, short-read RNA-seq has been widely applied in Acrididae functional genetic studies, although the transcripts must be assembled from short reads with assemblers such as Trinity^26^, SOAPdenovo-Trans^46^ or Trans-ABySS^47^. Due to the complex splicing mechanisms of eukaryotic cells, it is difficult to accurately reconstruct and reliably express transcriptomic isoforms with these short-read RNA-seq techniques. In contrast, PacBio sequencing can directly generate superlong transcripts with a short run time and without assembly, comprehensively solving these problems.

Here, we successfully performed the high-quality collection of transcripts from two Acrididae insects, *G. licenti* and *M. japonicus*, using PacBio sequencing. Compared with the de novo assembled unigenes obtained by RNA-seq, the length of the unigenes obtained by PacBio sequencing was greatly improved. This result shows that PacBio sequencing provides great advantages for obtaining complete transcripts and is beneficial for complex de novo transcriptome analyses. However, the drawbacks of PacBio sequencing were also obvious. Despite the vast amount of sequence data (*G. licenti*, 34.16 Gb clean data; *M. japonicus*, 34.55 Gb clean data), fewer unigenes were obtained by PacBio sequencing than by RNA-seq. This phenomenon has also been observed in other FL transcriptome studies^9,48,49^. One of the reasons could be attribute to the strict parameters setting in the process of FL transcript cluster.

As a key part of the transcriptional regulation system, TFs can affect the phenotypes of organisms by regulating downstream target genes and signaling networks. For example, the Hox protein Abdominal-A (Abd-A) can downregulate the expression of the cell cycle regulator gene *Cyclin E* (CycE) and the wing disc cuticle protein gene *WCP4*, which plays an important role in insect embryo development, somite differentiation and proleg formation^50,51^. Using the present data, 1,082 putative TFs from 41 TF gene families were identified in *G. licenti*, and 813 putative TFs from 39 TF gene families were identified in *M. japonicus*.

SSR markers have the characteristics of codominance, high reproducibility, abundant polymorphisms and easy detection, and they have therefore become an important genetic marker technology. SSR markers are often used to study the genetic differentiation, genetic diversity and population structure of species, which is of great significance for the protection of species, especially endangered species^52–54^. Here, we detected 11,840 and 10,814 SSRs from *G. licenti* and *M. japonicus*, respectively. These SSRs will serve as useful tools for analyzing genetic diversity, constructing genetic maps and investigating the population structures of *G. licenti* and *M. japonicus*.

LncRNAs are a group of RNA molecules with highly conserved secondary and tertiary structures, and their transcripts are generally longer than 200 nt. LncRNA can not only control gene expression through transcriptional and posttranscriptional regulation but also exert powerful biological functions by affecting protein localization and telomere replication. In recent years, a large number of lncRNAs have been identified from *Drosophila melanogaster*^55^, *Bombyx mori*^56^, *Plutella xylostella*^57^ and other insects, laying an important foundation for further studies of the functions of lncRNA in insect growth and development. To date, researchers studying lncRNAs in *D. melanogaster* have confirmed that lncRNAs can participate in many biological processes, such as sex determination, male courtship and X chromosome inactivation^58–61^. In this study, we predicted 829 lncRNAs in *G. licenti* and 706 lncRNAs in *M. japonicus* with PacBio sequencing, laying a foundation for the next step in studying the biological functions of lncRNAs in these two insects.

Our gene annotation results clearly showed that a large number of unigenes from *G. licenti* and *M. japonicus* can be classified for functional research. The NR annotation revealed that the species with the largest distribution of unigenes in *G. licenti* and *M. japonicus* was *Z. nevadensis* rather than more closely related *L. migratoria*, which may be caused by the incomplete genome assembly of *L. migratoria*^17^. But compared with published RNA-seq of the Acrididae species, unigenes obtained by PacBio sequencing has a higher proportion of annotation on the genomes of *L. migratoria*^18,19,62^. The KEGG analysis showed that 209 and 211 KEGG pathways were successfully annotated in *G. licenti* and *M. japonicus*, respectively. And among which Wnt, Notch, Hedgehog, Hippo and other pathways played critical roles in insect growth and development, for example, the Wnt signaling pathway has been shown to be essential for both embryogenesis and organogenesis, such as controlling axis elongation and leg development of one short-germ insect^63^, and the Hippo signaling pathway is an evolutionally-conserved signaling cascade that plays a role in controlling organ size during animal development, such as controlling the size of silkworm wings^64^. GO annotations are classified into three main categories: biological process, cellular component and molecular function. In this study, 8,065 and 7,637 unigenes of *G. licenti* and *M. japonicus* were respectively enriched in these three main categories. Among them, in the biological process category, 210, 178, 607 and 67 unigenes in *G. licenti* and 231, 188, 567 and 67 unigenes in *M. japonicus* were classified into the reproduction, reproductive process, developmental process, and growth subcategories, respectively. These genes provide important information for the study of growth and development, sex determination, fertilization, oviposition and other related processes in *G. licenti* and *M. japonicus*.

In conclusion, PacBio sequencing was performed on *G. licenti* and *M. japonicus*, and we provide here the first report of FL transcriptomes in Acrididae. The obtained FL transcriptome datasets enrich the data resources of *G. licenti* and *M. japonicus* and will provide support for future research on their functional genomics.

## Methods

### Sample collection and RNA preparation

Three adult females and three adult males of *G. licenti* and *M. japonicus* were collected from Yan’an City of Shaanxi Province, China. The whole body, except the gut, was immediately collected and stored in liquid nitrogen. According to the manufacturer's instructions, total RNA was extracted from each individual using TRIzol reagent (Invitrogen, Carlsbad, CA, USA), and RNA degradation and contamination were detected with 1% agarose gels. The integrity and purity of RNA were assessed by the Agilent 2,100 Bioanalyzer (Agilent Technologies, CA, USA) and NanoDrop 2000 (Thermo Scientific, Wilmington, DE, USA). Only RNAs with an RNA integrity number (RIN) score > 8.0 and 1.8 < OD260/280 < 2.2 were used for the preparation and construction pf PacBio and Illumina libraries.

### Library construction and sequencing

To construct the PacBio sequencing library, eligible RNAs from each individual were mixed in equal amounts and reverse-transcribed into FL cDNA using the SMARTer PCR cDNA Synthesis Kit (Clontech, CA, USA). The KAPA HiFi PCR Kits were used to amplify the FL cDNA by PCR, and the BluePippin Size Selection system (Sage Science, USA) was used to select the PCR products. The cDNA products with lengths of 1–6 kb were finally retained. After repairing the ends of the FL cDNA and connecting the SMRT dumbbell-type connector, the SMRTbell Template libraries were constructed using the SMRTbell Template Prep Kit. Agilent 2,100 Bioanalyzer and Qubit 2.0 (Life Technologies, Carlsbad, CA, USA) were used to evaluate the concentration and quality of these libraries. Finally, qualified libraries were sequenced using the PacBio Sequel platform (Pacific Biosciences, Menlo Park, CA, USA). Raw PacBio sequencing reads were stored in the Short Read Archive (SRA) of NCBI with accession number SRR10420895 for *G. licenti* and SRR10420906 for *M. japonicus*.

Six separate Illumina libraries were constructed for *G. licenti* and *M. japonicus* using the protocol of the Gene Expression Sample Prep Kit (Illumina, San Diego, CA, USA). Briefly, polyadenylated mRNA was isolated from total RNA using Oligo (dT) magnetic beads and then fragmented randomly with Fragmentation Buffer. The first-strand cDNA was synthesized with random hexamer primers using the fragmented mRNA as a template, and the second-strand cDNA was synthesized with DNA polymerase I (New England Biolabs) and RNase H (Invitrogen). After end repair, A-tail, adaptor ligation and purification with AMPure XP beads, PCR amplification was conducted. Finally, the six libraries were paired-end sequenced at 150 bp on Illumina HiSeq X Ten platform. Clean Illumina reads were produced after removing low-quality reads and adaptor reads, and then all clean reads from the same species were merged together for de novo assembled by using Trinity v2.5.1 software^26^ with the default parameters. Raw sequence data generated by Illumina were stored in the NCBI SRA with accession numbers (SRR10420893, SRR10420894, SRR10420900, SRR10420901, SRR10420903, SRR10420904) for *G. licenti* and (SRR10420896, SRR10420897, SRR10420898, SRR10420899, SRR10420902, SRR10420905) for *M. japonicus*.

### PacBio read error correction

The SMRT Link 5.1 pipeline from Pacific Biosciences^65^ was used for PacBio data processing. Briefly, CCS reads were extracted from raw reads with minFullPass = 1 and minPredictedAccuracy = 0.80. After discarding CCS reads with lengths shorter than 50 bp, the retained CCS reads were classified into FLNC and NFL transcripts according to whether they simultaneously contained 5′ primers, 3′ primers and poly-A tails. The ICE algorithm was used to cluster FLNC sequences to obtain consensus isoforms, and then, Arrow software (https://www.pacb.com/wp-content/uploads/SMRT_Tools_Reference_Guide_v600.pdf) was used to refine the consensus isoforms using the NFL to obtain polished consensus sequences. All polished consensus sequences were corrected using the Illumina RNA-seq short reads with the software Proovread v2.12^24^ with the default settings. To obtain the final transcriptome isoform sequences, the redundant sequences were removed by the CD-HIT software^66^, and the corrected consensus sequences were further screened. Due to the possible degradation of 5′ end sequences, isoforms from the same transcript were divided into different clusters, resulting in redundant sequences. Therefore, CD-HIT software was used to remove redundant sequences from the transcriptome isoform sequences and obtain the unigene sequences. Finally, BUSCO software^25^ with the insect lineage database (insecta_orthoDB9, created 13/02/2016) was used to assess the completeness of the unigenes.

### Coding sequences and transcription factor prediction

The coding sequence and corresponding protein sequence of the unigenes were predicted by the TransDecoder v5.0.1 package based on the ORF length, log-likelihood score and Pfam database protein domain sequences. The TFs were predicted from protein sequences by a prediction tool in an animal transcription factor database (AnimalTFDB)^67^.

### Simple sequence repeat prediction

An SSR is a set of repetitive DNA sequence with lengths varies from 2 base pairs to 13 base pairs and some motifs repeated 5–50 times, also known as a microsatellite^68^. MISA (v1.0; https://webblast.ipk-gatersleben.de/misa/)^30^ is a software used to identify SSRs, and through the analysis of transcript sequences, seven kinds of SSR can be identified: mononucleotides, dinucleotides, trinucleotides, tetranucleotides, pentanucleotides, hexanucleotides and compound SSRs. Only transcripts > 500 bp in length were subjected to SSR detection.

### Long noncoding RNA prediction

LncRNA is an important component of the transcriptome. Because lncRNA does not encode proteins, lncRNA can be obtained by screening transcripts for coding potential, judging whether they have coding potential or not, and filtering out the transcripts with coding potential. In this study, lncRNAs were predicted by screening the coding potential of transcripts using CPC^31^, CNCI^27^, CPAT^32^ and Pfam protein structure domain analysis, and select the intersection of four results as the set of lncRNAs.

### Identification of alternative splicing transcript isoforms

Due to the lack of annotated reference genome in *G. licenti* and *M. japonicus*, we used the all-vs-all BLAST method with high identity settings described by Liu et al. to de novo detecting AS transcript isoforms^69^. If the BLAST results meet the following conditions, it is considered as a candidate AS events: (a) the length of both sequences exceeded 1,000 bp, and the alignment contained two high-scoring Segment Pairs (HSPs); (b) the gap of AS exceeded 100 bp, and was located at least 100 bp from the 3′/5′ end; and (c) allowed a 5 bp overlap for all alternative scripts.

### Functional annotation

All unigenes were annotated using BLAST v2.2.31 software^27^ with the NR, Swiss-Prot, KOG, COG and eggNOG databases with cut-off E-value < 1E-5. According to the annotation results of the NR database, the Blast2GO v2.5 software^70^ was used to perform GO annotation. KEGG^33^ was annotated by KOBAS v2.0 software^71^, and Pfam was annotated by HMMER v3.1b2 software^72^.

## Supplementary information

Supplementary information 1.

Supplementary information 2.
