# Supplementary material for: Characterization and analysis of full-length transcriptomes from two grasshoppers, Gomphocerus licenti and Mongolotettix japonicus
Source: Sci Rep. 2020 Aug 26;10:14228. doi: 10.1038/s41598-020-71178-5 (PMC7450073; doi:10.1038/s41598-020-71178-5)
Supplement: Supplementary file 1 — Supplementary information 1. [file 41598_2020_71178_MOESM1_ESM.doc]

Supplemental Material for:

**Characterization and analysis of full-length transcriptomes from** **two grasshoppers, *Gomphocerus licenti* and** ***Mongolotettix japonicus***

Hao Yuan^1^, Xue Zhang^1^, Lina Zhao^1^, Huihui Chang^1^, Chao Yang^1,2^, Zhongying Qiu^3^, Yuan Huang^1^*

^1^ College of Life Sciences, Shaanxi Normal University, Xi’an 710062, China; yuanhao@snnu.edu.cn (H.Y.); zxsnowwww@gmail.com (X.Z.); 15529270320@163.com (L.Z.); chh127@snnu.edu.cn (H.C.)

^2^ Shaanxi Institute of Zoology, Xi’an, China; chaoy819@163.com (C.Y.)

^3^ School of Basic Medical Sciences, Xi’an Medical University, Xi’an, China; qiuzhongying11@126.com (Z.Q.)

* Corresponding author

E-mail: yuanh@snnu.edu.cn (YH)

Table S1 RNA-seq data output statistics in *G. licenti* and *M. japonicus* by Illumina platform.

|  | Read Number | | Base Number | | GC Content | | %≥Q30 | |
| --- | --- | --- | --- | --- | --- | --- | --- | --- |
| Species | *G. licenti* | *M. japonicus* | *G. licenti* | *M. japonicus* | *G. licenti* | *M. japonicus* | *G. licenti* | *M. japonicus* |
| male 1 | 22371991 | 22598983 | 6683261900 | 6762683128 | 45.87 | 46.94 | 94.66 | 93.57 |
| male 2 | 22821409 | 22429462 | 6822156836 | 6702533218 | 45.62 | 46.14 | 94.71 | 94.29 |
| male 3 | 21256557 | 21892971 | 6356771936 | 6550614104 | 45.27 | 46.16 | 94.45 | 94.58 |
| female 1 | 22128241 | 22660714 | 6618675342 | 6774059930 | 48.89 | 50.7 | 94.44 | 94.56 |
| female 2 | 29146965 | 23086319 | 8720182974 | 6904168334 | 48.6 | 49.18 | 94.77 | 95.17 |
| female 3 | 20919118 | 23861782 | 6257201358 | 7119623732 | 49.33 | 49.39 | 94.63 | 94.52 |
| All/Average | 138644281 | 136530231 | 41458250346 | 40813682446 | 47.26 | 48.09 | 94.61 | 94.45 |

Table S2 Using RNA-seq data to correct the low quality isoforms of PacBio sequencing by proovread software.

|  | *G. licenti* | | *M. japonicus* | |
| --- | --- | --- | --- | --- |
|  | low-quality | proovread | low-quality | proovread |
| Sequences | 601 | 595 | 544 | 536 |
| Total (bp) | 1,865,920 | 1,689,356 | 1,699,086 | 1,490,801 |
| Longest (bp) | 8,332 | 7,372 | 8,533 | 8,361 |
| Shortest (bp) | 127 | 505 | 187 | 505 |
| N50 (bp) | 3,672 | 3,429 | 3,610 | 3,340 |
| N75 (bp) | 2,929 | 2,731 | 2,792 | 2,483 |

Table S3 BUSCO analysis the completeness of *G. licenti* and *M. japonicus*.

| **Species** | ***G. licenti*** | ***M. japonicus*** |
| --- | --- | --- |
| Complete (C) | 70.4% (1167/1658) | 68.8% (1140/1658) |
| Complete and single-copy (S) | 48.0% (796/1658) | 47.2% (782/1658) |
| Complete and duplicated (D) | 22.4% (371/1658) | 21.6% (358/1658) |
| Fragmented (F) | 2.7% (45/1658) | 3.1% (51/1658) |
| Missing (M) | 26.9% (446/1658) | 28.1% (467/1658) |

Table S4 Length distribution and quality metrics of unigenes sequenced by Illumina.

| **Length Range** | ***Gomphocerus licenti*** | | ***Mongolotettix japonicus*** | |
| --- | --- | --- | --- | --- |
|  | Transcript | Unigene | Transcript | Unigene |
| 300-500 | 29,637(30.67%) | 21,796(40.78%) | 35,980(28.41%) | 22,396(41.74%) |
| 500-1000 | 24,688(25.55%) | 13,447(25.16%) | 29,762(23.50%) | 12,954(24.14%) |
| 1000-2000 | 20,776(21.50%) | 9,275(17.35%) | 27,835(21.98%) | 9,236(17.21%) |
| 2000+ | 21,540(22.29%) | 8,934(16.71%) | 33,066(26.11%) | 9,066(16.90%) |
| Total Number | 96,643 | 53,453 | 126,643 | 53,652 |
| Total Length | 136,517,140 | 62,761,737 | 199,205,336 | 63,097,752 |
| N50 Length | 2,371 | 2,047 | 2,671 | 2,074 |
| Mean Length | 1412.59 | 1174.15 | 1572.97 | 1176.06 |

Table S5 Comparison between unigenes sequenced by Illumina and PacBio.

|  | ***G. licenti*** | | ***M. japonicus*** | |
| --- | --- | --- | --- | --- |
|  | PacBio | Illumina | PacBio | Illumina |
| blast hit | 11192 (62.41%) | 7692 (14.39%) | 10632 (63.52%) | 7027 (13.10%) |
| no-blast hit | 6740 (37.59%) | 45761 (85.61%) | 6107 (34.48%) | 46625 (86.90%) |
| mapped *L. migratoria* | 8120 (45.28%) | 9695 (18.14%) | 8263 (49.36%) | 10773 (20.08%) |
